# Information Needs Assessment among Parents of Children with Cancer

**DOI:** 10.31557/APJCP.2019.20.6.1865

**Published:** 2019

**Authors:** Mohammad Esmail Motlagh, Mehdi Mirzaei-Alavijeh, Seyyed Nasrollah Hosseini

**Affiliations:** 1 *Department of Pediatrics, Faculty Medicine, Ahvaz Jundishapur University of Medical Sciences, Ahvaz, *; 2 *Social Development and Health Promotion Research Center, Kermanshah University of Medical Sciences, Kermanshah, *; 3 *Ministry of Health and Medical Education, Tehran, Iran. *

**Keywords:** Pediatrics, neoplasms, education, parents

## Abstract

**Background:**

Parents of children with leukemia should be receiving an extensive amount of information about the care of their child; the aim of this study was to determine the parents’ information needs of children with leukemia.

**Methods:**

A cross-sectional study design was used to describe medical, physical, mental and lifestyle information needs among parents of children with leukemia. 209 parents of children diagnosed with leukemia in the west of Iran, during winter 2018, voluntarily participated in individual interviews. Data were analyzed by SPSS version 16 using t-test, One-way ANOVA and bivariate correlations statistical tests at 95% significant level.

**Results:**

The mean age of participants was 39.45 years [95% CI: 38.35, 40.55], ranged from 27 to 58 years. Participants achieved 55.6% score of information needs. There was a significant relationship between higher education level (P< 0.001), better economic status (P=0.008) and more family size member (P=0.003) with information needs.

**Conclusion:**

Findings suggest that parents of children with leukemia need the information to learn how to take care of their childhood and could be useful for guiding implementers to planning and implement effective programs to promotion information of parents towards children with cancer.

## Introduction

In recent years, chronic diseases such as cancers have been one of the major health problems, and according to estimates by the world health organization, cancer incidence ratio could be increased annually to 15 million new cases in the year 2020 (Barnes et al., 2002). Cancer as a debilitating and prevalent disease is one of the main causes of child mortality (Panganiban-Corales et al., 2011), and it’s as a life-threatening event for children and their families (Ward et al., 2014). Leukemia is the most common cancer in children with accounting about 30% prevalence and due to its high morbidity, long hospitalization time, high treatment costs and psychological problems in the patient and his family has a heavy social burden (Shields et al., 2003) and reported the incidence ratio of both sexes is almost the same in Iran (Moussavi et al., 2014). Occurrence the Leukemia in children will be a critical situation for all family members (Ross and Olshan, 2004), and it’s a major effect on family member’s quality of life as well as children (Ozer et al., 2009). The children patients are one of the stressors in the family and parents should be able to deal with this situation (Amrock andWeitzman, 2014). Although in recent decades improvement in therapeutic methods has increased survival in children with Leukemia, but this increase in survival may expose the child to other health problems, such as decreased quality of life and consequently, this will cause stress and negative impact on the quality of life of parents as the main healthcare of these children (Litzelman, et al., 2011). Children with Leukemia need support from their parents to cope and deal with this situation (Trask et al., 2003). In addition children with Leukemia often have symptoms and complications that interact negatively with their parents’ relationship and disturb the child’s compliance (O’Conner-Von, 2009). Studies have shown that parents’ well-informed participation facilitates children’s health care, thus, parents need the knowledge and skills to care of children (Jordan et al., 2008; Sahler et al., 2005). Several studies indicated information towards diagnosis, treatment and complications can be reducing parental disturbances, create a normal family environment, reduce anxiety and increase control in parents (Wong et al., 2006; Soanes et al., 2009; Blaauwbroek et al., 2007; Ressler et al., 2003). It also empowers parents to follow up on their children’ care (Yuan, 2011). For this reason, parents of children with leukemia need the information to manage and solve their children’s problems; in other hand, health information is one of the strategies of health care organizations to promote patient care and the participation of families in the care and treatment process of the disease (Hummelinck and Pollock). In this regards, studies suggest that the first step to planning a health promotion program understanding the existing situation (Mirzaei-Alavijeh et al., 2018). The aim of this study was to determine the parents’ information needs of children with leukemia.

## Materials and Methods 

*Participants and Procedure*

A current cross-sectional study was carried out on 209 parents of children with leukemia referred to Dr. Mohammad Kermanshahi Hospital in Kermanshah city, the west of Iran, during winter 2018.

*Sampling*

The required sample size is calculated using the following formula:

$n=\frac{\sigma^{2}*z_{1-\frac{a}{2}}^{2}}{d^{2}}$

The sample size was calculated at 95% significant level according to the results of a pilot study and a sample of 209 was estimated. The sampling method was simple sampling and participants were selected among parents of children with leukemia referred to Dr. Mohammad Kermanshahi Hospital, in the west of Iran. 

*Data Gathering*

Participants filled out a questionnaire by interview including the background variables and information needs components.

*Measures*

Prior to conducting the main study, a pilot study was carried out. Initially, the relevant questionnaires were administered to 20 parents of children with leukemia who were similar to the study population in order to estimate the duration of the study conduction and to evaluate the reliability of the questionnaire. Estimated reliability using alpha Cronbach coefficient for each information needs components questionnaire were as follows: medical information (α=0.81); physical care information (α= 0.88); mental care information (α= 0.89); lifestyle information (α = 0.85). As well as, the reliability of the total questionnaire was 0.92.

*Backgrounds Variables*

The variables assessed in this study included: age (years), age of children (years), years of diseases diagnosis (years), education level (under diploma/diploma/academic), sex (men/women), children sex (girl/boy), economic status (weak, average, good), insurance (yes/no), and family member size (three /four and five /more than five).

*Information Needs Scale*

Information needs scale was designed based on a standard questionnaire (Borjalilu et al., 2017; Aydinok et al., 2005; Ringnér et al., 2011) and included 13 items under four constructs including (a) medical information; (b) physical care information; (c) mental care information; (d) lifestyle information. Three items were designed to measure lifestyle information (e.g., How much you know about treatment methods of your child’s disease). Three items were designed to measure physical care information (e.g., How much you know about pain relief management methods of your child). Four items were designed to mental care information (e.g., How much you know about coping methods with fear and anxiety in your child). Three items were designed to evaluate lifestyle information (e.g., How much you know about the needs of having relatives after your child’s illness). In order to facilitate participants’ responses to the items, all items were standardized to a 5-point Likert scale, ranging from 1 (very low) to 5 (very much). 

*Statistical Analysis*

Data were analyzed by SPSS version 16 using appropriate statistical tests including bivariate correlations, Independent Samples t-test, and One-Way ANOVA at 95% significant level. Bivariate correlations were computed to the direction of the associations between the information needs constructs scores. Independent Samples t-test and One-Way ANOVA were performed to explain the relationship between the backgrounds variables and information needs constructs scores. Alpha Cronbach coefficient was used to estimate the internal consistency of the various measures.

*Inclusion and Exclusion Criteria*

Only the subjects who had children with leukemia were eligible to participate in this study. Subjects who did not answer all the questions were excluded from the analysis.

*Ethical Approve*

The Research Ethics Board of the Ahvaz Jundishapur University of Medical Sciences approved the study protocol (IR.AJUMS.REC.1397.439). Further, the subjects had been given the information statement. Individual personal information was kept confidential.

## Results

The mean age of participants was 39.45 years [95% CI: 38.35, 40.55], ranged from 27 to 58 years. The mean age of children was 5.57 years [95% CI: 5.22, 5.92], ranged from 1 to 10 years. The mean years of diseases diagnosis among children 2.28 years [95% CI: 2.13, 2.43], ranged from 1 to 5 years. More details of the background variables of the respondents are shown in Table 1.

The mean, standard deviation, maximum score acquirable of 100%, as well as the correlation of the various dimensions information needs were determined. All the different dimensions represented a significant statistical correlation of 0.01 (Table 2).

The relationship between background variables and the various dimensions of information needs was showed in Table 3. Our results indicated educational level was associated significantly and statistically with the dimensions of medical information, physical care information, mental care information and information needs total. As well as, the economic status associated significantly and statistically with the dimensions of medical information, physical care, and information needs total. In addition, family member size was associated significantly and statistically with the dimensions of physical care information, lifestyle information and information needs total.

**Table 1:** Distribution of the Background Variables among the Respondents

Variables	Number	Percent
Parent Sex		
Female	125	66.8
Male	62	33.2
Children Sex		
Female	88	47.1
Male	99	52.9
Education level		
Under diploma	66	35.3
Diploma	99	52.9
Academic	22	11.8
Economic status		
Weak	46	24.6
Average	84	44.9
Good	57	30.5
Insurance		
Yes	155	82.9
No	32	17.1
Family Member Size		
Three	72	38.5
Four and five	97	51.9
More than five	18	9.6

**Table 2:** Mean, Standard Deviation and Correlation of the Various Dimensions of Information Needs

	Medical	Physical care	Mental care	Life style	Mean (SD)	Score range	Maximum score acquirable of 100%
Medical care	1				9.01 (1.78)	3-15	60.06%
Physical care	0.723**	1			9.07 (1.66)	3-15	60.46%
Mental care	0.506**	0.444**	1		10.56 (2.69)	4-20	52.80%
Life style	0.505**	0.406**	0.798**	1	7.50 (2.10)	3-15	50.00%
Information needs total	0.793**	0.741**	0.879**	0.858**	36.15 (6.81)	13-65	55.61%

**Table 3:** Relationship between Background Variables and the Various Dimensions Information Needs

Variables		Medical	Physical care	Mental care	Life style	Information needs total
Parent Sex	Female	8.97 (1.58)	9.00 (1.61)	10.54 (2.68)	7.43 (2.09)	35.96 (6.67)
	Male	9.08 (2.12)	9.20 (1.75)	10.59 (2.71)	7.66 (2.14)	36.54 (7.12)
	Statistical Test +	-0.378	-0.781	-0.126	-0.699	-0.555
	P	0.706	0.436	0.9	0.485	0.58
children Sex	Female	9.14 (1.64)	9.10 (1.69)	10.78 (3.14)	7.70 (2.45)	36.73 (7.43)
	Male	8.88 (1.88)	9.05 (1.63)	10.36 (2.21)	7.33 (1.73)	36.63 (6.20)
	Statistical Test +	0.994	0.212	1.046	1.179	1.1093
	P	0.322	0.832	0.297	0.24	0.276
Education level	Under diploma	8.15 (2.22)	8.53 (1.95)	10.13 (3.08)	7.18 (2.36)	34.01 (7.95)
	Diploma	9.80 (1.11)	9.21 (1.30)	10.54 (2.42)	7.55 (1.99)	36.62 (5.58)
	Academic	10.22 (1.63)	10.09 (1.60)	11.90 (2.15)	8.27 (1.60)	40.50 (5.85)
	Statistical Test +	16.701	8.664	3.69	2.294	8.675
	Significance	< 0.001	< 0.001	0.027	0.104	< 0.001
Economic status	Weak	8.39 (2.66)	8.54 (2.30)	10.34 (3.16)	7.08 (2.44)	34.00 (8.86)
	Average	8.80 (1.14)	8.98 (1.04)	10.41 (2.91)	7.42 (2.29)	37.50 (6.05)
	Good	9.80 (1.35)	9.63 (1.4)	10.94 (1.77)	7.96 (1.34)	37.51 (5.36)
	Statistical Test +	9.913	5.976	0.852	2.349	4.983
	Significance	< 0.001	0.003	0.428	0.098	0.008
Insurance	Yes	9.04 (1.70)	9.09 (1.57)	10.64 (2.74)	7.62 (2.12)	36.40 (6.66)
	No	8.84 (2.13)	9.00 (2.04)	10.15 (2.41)	6.93 (1.96)	34.93 (7.49)
	Statistical Test +	0.582	0.279	0.936	1.69	1.111
	Significance	0.561	0.78	0.351	0.093	0.268
Family Member Size	Three	8.79 (2.01)	8.45 (1.76)	10.00 (2.01)	6.75 (1.93)	34.00 (6.66)
	Four and five	9.20 (1.39)	9.51 (1.54)	10.86 (2.94)	7.91 (1.92)	37.50 (6.23)
	More than five	8.83 (2.47)	9.16 (0.92)	11.16 (3.32)	8.33 (2.33)	37.51 (8.43)
	Statistical Test +	1.225	9.139	2.693	8.497	6.185
	Significance	0.296	< 0.001	0.07	< 0.001	0.003

+, t-student test; ++, ANOVA test

## Discussion

The aim of this study was to identify parents’ information needs of children with leukemia. The results of our study indicated that the participants achieved 60.06%, 60.46%, 52.80 and 50% of score medical, physical, mental and lifestyle information, respectively. In addition, the findings of the current study suggest that among the background variables three variables were significantly related to the information needs among the participants: 1) education level, 2) economic status and 3) family size member.

Our findings indicated participants achieved 60.06% of score medical care information. Several studies have shown that medical needs are one of the most essential information needs of parents of a child with cancer. In this regards, Maree et al in their study among parents of children with cancer treated at an academic hospital in the Gauteng province of South Africa and reported parents are seeking information on the disease and therapeutic methods (Maree et al., 2016). In addition, Pyke-Grimm carried out a cross-sectional survey among 58 parents who had a child less than 13 years of age diagnosed with cancer and reported the treatments and tests were the highest information needs among them (Pyke-Grimm, 1999). As well as, Christiansen et al carried out research in two pediatric oncology centers in the UK with aim of determined problems, perceptions and information needs of parents and carers regarding oral chemotherapy and indicated parents/carers area need improvement (Christiansen et al., 2008). If parents are aware of their child medical needs can lead to increases quality care of their child’s, accordingly, it is necessary to increase parents medical information in order to the promotion of child quality of life.

Another information needs investigated in our study was physical care information. Several studies have shown that one of the activities of parents during childbirth is physical care (Holm et al., 2006; Macnab et al., 2000). One of the information need in physical care dimension was how to care for children when their child experiences pain. In this regards the results of studies have shown that parents are through appropriate interventions such as cognitive behavioral therapy and communication skills help to reduce the perception of pain in their children (Palermo et al., 2010; Palermo et al., 2009). Furthermore, another one of the information needs investigated in physical care dimension was about nutrition. Studies have shown parents have used of different ways to feed their child such as threatening, pushing and begging which are often not correct (Bauer et al., 2011; Demark-Wahnefried and Jones, 2008; Didehbani et al., 2011; Fleming et al., 2015). Considering that the participants in this study did not receive about 40% of the physical care dimension, it seems that planning programs to improve physical care information is necessary.

Information needs for mental health were another dimension of information that was studied in our research. This dimension shows that parents of children with leukemia seek information about their child’s excitement such as fear, anxiety, and anger. Cancer of children due to impairment in daily functioning, complications of treatment, lack of knowledge about the disease and fear of death are stressful for children and their parents (Rodriguez et al., 2011). Our results indicated participants received approximately half the maximum achievable scores for this dimension. In line with our findings, Kristensson-Hallstrom and Elander (1997) believed that parents are the intermediary between the sick child and the treatment team, which reduces the child’s anxiety. As well as, Starke and Möller (2002) reported a sense of feel being involved in childcare can lead to improving the care of children by parents.

The lifestyle needs information was another dimension of studied in our research. Some items that were examined in this dimension included communicating with the wife/husband, communicate with other children’s and communicate with relatives. Our findings indicated that the participants’ information in this dimension was lower than the other dimensions examined Norberg and Boman, (2013) in their study pointed out that childhood cancer as posttraumatic stress disorder which causing disruptions in the performance of the spouses and they need the information to control the conditions. Our results provide important information that may be used to planning healthy lifestyle promotion programs among parents’ of children with leukemia. These results can be used by health educator in Iran, and it seems to focus on health educator should be to promotion of healthy lifestyle among parents’ of children with leukemia.

Our findings showed that participants with higher education significantly had a higher mean score for their information needs which indicated that they had fewer information needs. This result is consistent with other studies, and several studies show a direct and significant relationship between parental education and child care (Cleland and Van Ginneken, 1988; Imdad et al., 2011; Grépin and Bharadwaj, 2015). Furthermore, family member size including four and more significantly had less information needs. This was consistent with findings by other studies. For example, Noohi et al., (2016) reported the effective role of family support in carrying out health-related behaviors. Our study also showed that better economic status is often combined with the higher mean score for their information needs which indicated that they had fewer information needs among the participants. This result is in line with the findings of earlier studies investigating the children health care (Benzeval et al., 2000).

The findings reported in this study have certain limitations. A major limitation of our study that was investigated only four types of information care needs and other needs (such as, financial, travel needs and home support) by parents has not been investigated. Second, the information is based on interview, which always faces the risk of recall bias. Third, data collection only among sample of parents of children with leukemia in the west of Iran and due to non-probability nature of sampling, results cannot be generalized to other population of parents of children with cancer. Finally, the high rejection rate is another limitation of our study.

In conclusion, the present study provides useful information that may be useful in planning intervention programs to improve the information of Iranian parents about medical care, physical care, mental care and lifestyle care for children with cancer. We found that participants received half the maximum achievable scores for information needs. Based on our results, development and implementing of educational programs in order to enhance the information needs of parents of children with cancer is essential.

## Conflict of interest

The authors declared no conflict of interests.

## References

[B1] Amrock SM, Weitzman M (2014). Parental psychological distress and children’s mental health: results of a national survey. Acad Pediatr.

[B2] Aydinok Y, Erermis S, Bukusoglu N, Yilmaz D, Solak U (2005). Psychosocial implications of thalassemia major. Pediatr Int.

[B3] Barnes J, Kroll L, Lee J (2002). Factors predicting communication about the diagnosis of maternal breast cancer to children. J Psychosom Res.

[B4] Bauer J, Jürgens H, Frühwald MC (2011). Important aspects of nutrition in children with cancer. Adv Nutr.

[B5] Benzeval M, Taylor J, Judge K (2000). Evidence on the relationship between low income and poor health: is the government doing enough?. Fisc Stud.

[B6] Blaauwbroek R, Zwart N, Bouma M (2007). The willingness of general practitioners to be involved in the follow-up of adult survivors of childhood cancer. J Cancer Surviv.

[B7] Borjalilu S, Sharif Z, Sabbagh-Bani-Azad M (2017). The information needs of parents of children with cancer: A qualitative study. J Qual Res Health Sci.

[B8] Christiansen N, Taylor KM, Duggan C (2008). Oral chemotherapy in paediatric oncology in the UK: problems, perceptions and information needs of parents. Pharm World Sci.

[B9] Cleland JG, Van Ginneken JK (1988). Maternal education and child survival in developing countries: the search for pathways of influence. Soc Sci Med.

[B10] Demark-Wahnefried W, Jones LW (2008). Promoting a healthy lifestyle among cancer survivors. Hematol Oncol Clin North Am.

[B11] Didehbani N, Kelly K, Austin L, Wiechmann A (2011). Role of parental stress on pediatric feeding disorders. Child Health Care.

[B12] Fleming CA, Cohen J, Murphy A (2015). Parent feeding interactions and practices during childhood cancer treatment. A qualitative investigation. Appetite.

[B13] Grépin KA, Bharadwaj P (2015). Maternal education and child mortality in Zimbabwe. J Health Econ.

[B14] Imdad A, Yakoob MY, Bhutta ZA (2011). Impact of maternal education about complementary feeding and provision of complementary foods on child growth in developing countries. BMC Public Health.

[B15] Jordan A, Eccleston C, Crombez G (2008). Parental functioning in the context of adolescent chronic pain: a review of previously used measures. J Pediatr Psychol.

[B16] Kristensson-Hallstrom I, Elander G (1997). Parents’ experience of hospitalization: different strategies for feeling secure. Paediatr Nurs.

[B17] Litzelman K, Catrine K, Gangnon R, Witt WP (2011). Quality of life among parents of children with cancer or brain tumors: the impact of child characteristics and parental psychosocial factors. Qual Life Res.

[B18] Holm KE, Patterson JM, Gurney JG (2003). Parental involvement and family-centered care in the diagnostic and treatment phases of childhood cancer: results from a qualitative study. J Pediatr Oncol Nurs.

[B19] Hummelinck A, Pollock K (2006). Parents’ information needs about the treatment of their chronically ill child: a qualitative study. Patient Educ Couns.

[B20] Macnab A J, Thiessen P, McLeod E, Hinton D (2000). Parent assessment of family-centered care practices in a children’s hospital. Child Health Care.

[B21] Maree JE, Parker S, Kaplan L, Oosthuizen J (2016). The information needs of south african parents of children with cancer. J Pediatr Oncol Nurs.

[B22] Mirzaei-Alavijeh M, Ahmadi-Jouybari T, Vaezi M, Jalilian F (2018). Prevalence, cognitive and socio-demographic determinants of prostate cancer screening. Asian Pac J Cancer Prev.

[B23] Moussavi F, Hosseini SN, Saket S, Derakhshanfar H (2014). The First CBC in Diagnosis of childhood acute lymphoblastic leukemia. Int J Med Invest.

[B24] Noohi E, Peyrovi H, Goghary ZI, Kazemi M (2016). Perception of social support among family caregivers of vegetative patients: A qualitative study. Conscious Cogn.

[B25] Norberg AL, Boman KK (2013). Mothers and fathers of children with cancer: loss of control during treatment and posttraumatic stress at later follow-up. Psychooncology.

[B26] O’Conner-Von S (2009). Coping with cancer: A Web-based educational program for early and middle adolescents. J Pediatr Oncol Nurs.

[B27] Ozer ZC, Firat MZ, Bektas HA (2009). Confirmatory and exploratory factor analysis of the caregiver quality of life index-cancer with Turkish samples. Qual Life Res.

[B28] Palermo TM, Eccleston C, Lewandowski AS, Williams AC, Morley S (2010). Randomized controlled trials of psychological therapies for management of chronic pain in children and adolescents: an updated meta-analytic review. Pain.

[B29] Palermo TM, Wilson AC, Peters M, Lewandowski A, Somhegyi H (2009). Randomized controlled trial of an Internet-delivered family cognitive–behavioral therapy intervention for children and adolescents with chronic pain. Pain.

[B30] Panganiban-Corales AT, Medina MF (2011). Family resources study: part 1: family resources, family function and caregiver strain in childhood cancer. Asia Pac Fam Med.

[B31] Pyke-Grimm KA, Degner L, Small A, Mueller B (1999). Preferences for participation in treatment decision making and information needs of parents of children with cancer: a pilot study. J Pediatr Oncol Nurs.

[B32] Ressler IB, Cash J, McNeill D, Joy S, Rosoff PM (2003). Continued parental attendance at a clinic for adult survivors of childhood cancer. J Pediatr Hematol Oncol.

[B33] Ringnér A, Jansson L, Graneheim UH (2011). Parental experiences of information within pediatric oncology. J Pediatr Oncol Nurs.

[B34] Ross JA, Olshan AF (2004). PPediatric cancer in the United States: the children’s oncology group epidemiology research program. Cancer Epidemiol Biomarkers Prev J.

[B35] Rodriguez EM, Dunn MJ, Zuckerman T (2011). Cancer-related sources of stress for children with cancer and their parents. J Pediatr Psychol.

[B36] Sahler OJZ, Fairclough DL, Phipps S (2005). Using problem-solving skills training to reduce negative affectivity in mothers of children with newly diagnosed cancer: report of a multisite randomized trial. J Consult Clin Psychol.

[B37] Shields L, Kristensson-Hallström I, O’callaghan M (2003). An examination of the needs of parents of hospitalized children: comparing parents’ and staff’s perceptions. Scand J Caring Sci.

[B38] Soanes L, Hargrave D, Smith L, Gibson F (2009). What are the experiences of the child with a brain tumour and their parents?. Eur J Oncol Nurs.

[B39] Starke M, Möller A (2002). Parents’ needs for knowledge concerning the medical diagnosis of their children. J Child Health Care.

[B40] Trask PC, Paterson AG, Trask CL (2003). Parent and adolescent adjustment to pediatric cancer: associations with coping, social support, and family function. J Pediatr Oncol Nurs.

[B41] Ward E, DeSantis C, Robbins A, Kohler B, Jemal A (2014). Childhood and adolescent cancer statistics, 2014. CA Cancer J Clin.

[B42] Wong MYF, Chan SWC (2006). The qualitative experience of Chinese parents with children diagnosed of cancer. J Clin Nurs.

[B43] Yuan B (2011). Outreach strategies for expanding health insurance coverage in children. J Evid Based Med.

